# 

**Published:** 1879-12

**Authors:** 


### CONTENTS:

ARTICLE I.—Proceedings of the Maryland and District of Columbia Dental Society. (Continued.) .... 337
ARTICLE II.—Transactions of the Odontological Society of Great Britain. (Continued.) 353
ARTICLE III.—A National Dental Association. By Prof. R. B. Winder .... 359
ARTICLE IV.—Strengthening a Weak Tooth by Heavy Gold and Screws, etc. By Dr. Geo. A. Mills .... 366
ARTICLE V.—Consumption; Its Treatment, etc. By James H Salisbury, M. D. .... 371
ARTICLE VI.—A Singular Case.—Extrusion of a Bicuspid. By Prof. Hodgkin .... 376

### EDITORIAL, ETC.

The Salisbury Treatment of Consumption .... 377
Metallurgy and Chemistry of Metals .... 378

### BIBLIOGRAPHICAL.

Treatise on Dental Caries. By Dr. Magitot. Translated by Dr. Thos. E. Chandler .. 379
American Academy of Dental Science .... 382

### MONTHLY SUMMARY

Artificial Palate .... 383
The Relation between Age of Mother and Sex of Child .... 383
Steel Rust .... 383
Nickel Plating without a Battery .... 384
Tobacco Smoke .... 384
Sugar and Teeth .... 384
Artificial India Rubber .... 384

